# Basal Cell Carcinoma of the External Genitalia: A Population-Based Analysis

**DOI:** 10.3389/fonc.2020.613533

**Published:** 2021-01-26

**Authors:** Xi Chen, Yulong Hou, Can Chen, Guan Jiang

**Affiliations:** ^1^ Department of Dermatology, Affiliated Hospital of Xuzhou Medical University, Xuzhou, China; ^2^ Hebei Medical University, Shijiazhuang, China

**Keywords:** basal cell carcinoma, genitalia (male and female), SEER, overall survival, disease specific survival

## Abstract

**Introduction:**

Basal cell carcinoma (BCC) located on the genitalia is rare; data on the clinicopathologic features and survival outcomes are only available through case reports and small case series studies.

**Purpose:**

This study aimed to explore the epidemiology and identify the prognostic factors of genital BCCs.

**Methods:**

We queried the 18 registries of the Surveillance, Epidemiology, and End Results database for patients with primary BCCs of the genital skin from 2000 through 2017. The primary endpoint was overall survival (OS) and disease specific survival (DSS). Kaplan-Meier survival analysis was conducted to assess the impact of clinicopathological variables on OS and DSS. Multivariate Cox proportional hazards model was performed to evaluate risk factors for OS.

**Results:**

A total of 1,607 cases of genital BCCs were identified. The cohort was composed of 1,352 women (84.1%) and 255 men (15.9%). The median (P25, P75) age of the entire cohort was 73(63–82)years. White patients accounted for 87.2% of the cases. For women and men, the most common site of involvement was the labia majora (89.6%) and scrotum (74.5%), respectively. The majority of patients with genital BCC had localized disease (75.5%). Kaplan-Meier survival analysis showed that female genital BCCs experienced better DSS than men (209.1 months vs 194.8 months); for men, BCCs located on the scrotum had better DSS and OS than those on the penis (P < 0.05 for both endpoints). All patients with distant disease died of disease-specific death, and the average survival time was 8.2 months. Multivariate analysis revealed that age, primary site, and stage were independent determinants of OS for men, while tumor size, histologic subtype, and race were not. For women, factors associated with worse OS included increasing age, tumor size more than 2 cm, and distant disease; factors associated with a decreased risk included “other” and “unknown” races.

**Conclusion:**

The prognosis of genital BCCs is excellent, while the survival of distant disease is very poor. Despite similar clinicopathologic features and overall survival outcomes, men and women should be treated as two different entities when making survival predictions.

## Introduction

Basal cell carcinoma (BCC) is the most common malignancy and accounts for 75% non-melanoma skin cancers (1, 2). Ultraviolet (UV) radiation is considered the prime factor in its pathogenesis; unsurprisingly, more than 80% of BCCs are located on the sun-exposed skin of the elderly, especially in the head and neck regions (3, 4). BCCs can also involve areas with less sun exposure such as the trunk (3). However, only rare BCCs occur on sun-protected sites, and genital BCCs account for less than 1% of all BCCs, according to published literature (2, 4).

Given the extremely rare occurrence of genital BCCs, most of the records on genital BCCs are single cases or small case series, which most likely represent the extreme situation in clinical practice and lack of universality. To our knowledge, no comprehensive study on genital BCCs has performed a direct comparison between women and men, as well as between genital subsites *via* Kaplan–Meier analysis and multivariate analysis. Herein, detailed analyses were conducted utilizing the data from the Surveillance, Epidemiology, and End Results (SEER) program database. This analysis will provide important counseling value in clinical practice.

## Methods

### Patient Selection

We queried the SEER database’s 18 registries for patients diagnosed with primary BCCs of the external genitalia between the years 2000 and 2017. The SEER database contains epidemiologic information on cancer survival and is updated annually by the National Cancer Institute, covering 28% of the US population. The right to access these data through SEER Stat software was granted by the National Cancer Institute after submitting the signed SEER data-use agreement. Institutional review board approval was not necessary for this study due to publicly accessible data. Patients with genital BCC were identified using the histologic codes 8090/3 (basal cell carcinoma and not otherwise specified [NOS]), 8091/3 (multifocal superficial basal cell carcinoma), 8092/3 (infiltrating basal cell carcinoma, NOS), 8093/3 (basal cell carcinoma, fibroepithelial), and 8097/3 (basal cell carcinoma, nodular), as well as the primary site codes C51.0 (labium majus), C51.1 (labium minus), C51.2 (clitoris), C51.8 (overlapping lesion of vulva), C51.9 (vulva, NOS), C60.0 (prepuce), C60.2 (body of penis), C60.8 (overlapping lesion of penis), C60.9 (penis, NOS), and C63.2 (scrotum, NOS). Patients with BCC but without histologic confirmation were excluded.

### Variables

The following variables were extracted for analysis: sex, age, race, year of diagnosis, primary site, tumor size, histologic type, stage, cause of death, survival months, and vital status. According to the SEER Historic Stage A, stage was coded as localized (confined to the boundary of the primary organ), regional (direct extension to adjacent organs or structures or with regional lymph node involvement), and distant (spread to distant parts of the primary tumor). Race was grouped into the categories of white, black, and others (American Indian/Alaska Native and Asian/Pacific Islander). Tumor size was determined by comprehensive analyses of “CS tumor size (2004–2015)” and “Tumor Size Summary (2016+)”. For the sake of this analysis, the continuous variable age was categorized as follows (years): ≤60, 61–70, 71–80, or >80, and tumor size was categorized into two groups (cm): ≤2 or >2. For convenience, body of penis, overlapping lesion of penis, prepuce, and penis NOS were reclassified as penis. Men were divided into the penis group and the scrotum group by anatomic site. Women were divided into the following subgroups based on the location records of the SEER database: labium majus, labium minus, clitoris, overlapping lesion of vulva, and vulva NOS. Histopathological subtypes were classified in accordance with the World Health Organization criteria, including superficial, nodular, infiltrating, fibroepithelial, and BCC NOS.

### Statistical Analysis

Differences in clinicopathologic characteristics between women and men were compared using the Chi-squared tests (categorical variables) and Student’s t-test (continuous variables). The primary endpoints were overall survival (OS, defined as the time from diagnosis to death from any cause) and disease specific survival (DSS, defined as the time from diagnosis to a documented death due to BCC). Kaplan–Meier survival analysis was conducted to assess the impact of various variables on OS and DSS (univariate analysis), and the statistical difference in curves was calculated by log-rank tests. Age, gender, and covariates with log-rank P < 0.2 were chosen for multivariate analysis. Through Cox proportional hazard models, multivariate analysis was performed to identify important predictors for survival. All statistical analyses were performed using SPSS version 26.0. Two-sided P < 0.05 was considered statistically significant.

## Results

### Clinicopathologic Characteristics

A total of 1,607 patients diagnosed with genital primary BCCs from 2000 to 2017 were identified and included in this study. The clinicopathologic characteristics and demographics data of patients are summarized in **Tables 1** and **2**, respectively. This cohort was composed of 1,352 women (84.1%) and 255 men (15.9%). The female to male ratio was 5.3. Patients over 85 years in the SEER database were simply classified as 85+. A total of 307 cases over 85 years were included in this study and considered 85 years in the average age estimate for this cohort. The age of women ranged from 20 years to 85+ years (median 73 years) and man from 34 years to 85+ years (median 71 years). Of the cohort, 87.2% were white and 75.5% had localized disease. The mean tumor size of men was 1.59 cm (range 0.2–6.0 cm), similar to that of women (mean 1.79 cm; range 0.1–9.5cm). Among the patients with known histologic subtypes, most presented with nodular subtype (84.8%); others presented with superficial (6.3%), infiltrative (4.1%), and fibroepithelioma (4.8%) subtype. A total of 347 women had specific lesion sites, with the labia majora being the most common involvement (89.6%). Most male genital BCCs presented in the scrotum (74.5%).

**Table 1:** Clinicopathologic characteristics of patients with genital basal cell carcinoma by sex.

Characteristics	Female (n = 1352)	Male (n = 255)	P-value
Age(years)			
Median(P25, P75)	73 (63, 82)	71 (61, 81)	0.059
Range	20–85+	34–85+	0.127
≤60	292 (21.6%)	55 (21.6%)	
61–70	279 (20.6%)	69 (27.1%)	
71–80	372 (27.5%)	64 (25.1%)	
>80	409 (30.3%)	67 (26.3%)	
Race			0.026
White	1,187 (87.8%)	215 (84.3%)	
Black	40 (2.9%)	6 (2.4%)	
Others	79 (5.8%)	15 (5.9%)	
Unknown	46 (3.4%)	19 (7.5%)	
Tumor size(cm)			
Mean ± SD	1.8 ± 0.52	1.6 ± 1.29	0.166
Range	0.1–9.5	0.2–6	0.001
≤2	457 (33.8%)	67 (26.3%)	
>2	174 (12.9%)	21 (8.2%)	
Unknown	721 (53.3%)	167 (65.5%)	
Primary site			
Labium majus	311 (23%)	–	
Labium minus	18 (1.3%)	–	
Clitoris	8 (0.6%)	–	
Overlapping lesion of vulva	10 (0.7%)	–	
Vulva NOS	1,005 (74.3%)	–	
Penis	–	65 (25.5%)	
Scrotum	–	190 (74.5%)	
Histopathology			0.056
Superficial	24 (1.8%)	5 (2.0%)	
Nodular	321 (23.7%)	69 (27.1%)	
Infiltrative	15 (1.1%)	4 (1.6%)	
Fibroepithelioma	14 (1%)	8 (3.1%)	
Basal cell carcinoma NOS	978 (72.3%)	169 (66.3%)	
SEER Historic Stage A			0.175
Localized	1,029 (76.1%)	184 (72.2%)	
Regional	30 (2.2%)	4 (1.6%)	
Distant	3 (0.2%)	2 (0.8%)	
Unknown	290 (21.4%)	65 (25.5%)	

**Table 2:** Demographics data of patients.

Demographics	N	%
Total	1,607	100%
Race		
White	1,402	87.2%
Black	46	2.9%
American Indian/Alaska Native	7	0.4%
Asian/Pacific Islander	87	5.4%
Unknown	65	4.0%
Sex		
Female	1,352	84.1%
Male	255	15.9%
Age		
20–30 years	7	0.4%
31–40 years	32	2.0%
41–50 years	87	5.4%
51–60 years	221	13.8%
61–70 years	348	21.7%
71–80 years	436	27.1%
81–84 years	169	10.5%
85+ years	307	19.1%

### Survival Analysis by Sex, Age, and Race

**Figure 1:**
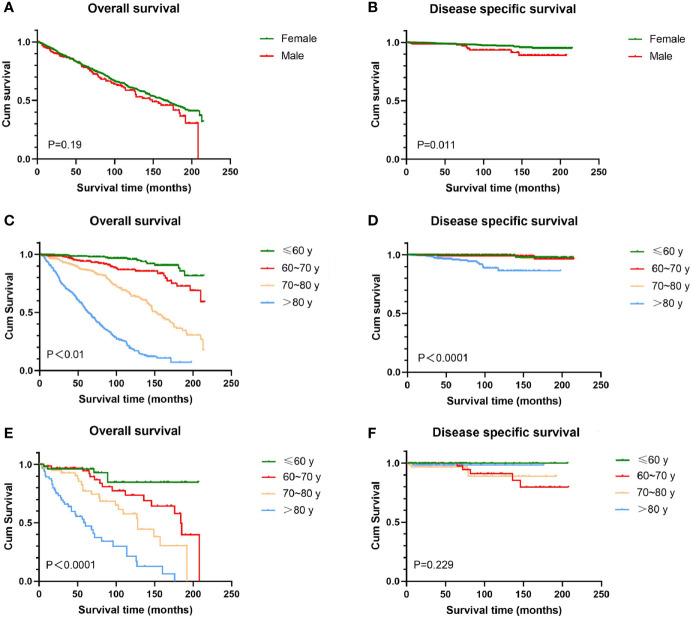
Kaplan-Meier estimates of OS and DSS by sex and age. There was no significant difference in OS between female and male **(A)**, while female experienced higher DSS than men **(B)**. OS decreased as age increased irrespective of sex **(C, E)**. Female over 80 years had significantly worse DSS than other age groups **(D)**; for male, difference in DSS between ages does not reach significance **(F)**. OS, overall survival; DSS, disease-specific survival; BCC, basal cell carcinoma.

There was no significant difference in OS between women and men, and the overall 5-year survival rates in this study were 79.5 and 77.8%, respectively. Women experienced higher DSS than men (209.1 months *vs* 194.8 months, P = 0.011). Regardless of gender, OS decreased as age increased (P < 0.0001), which is not a surprising finding considering the older age composition of this study. Women with age over 80 years had significantly worse DSS than other age groups (P < 0.0001), while there was less prognostic value in age for male DSS (P = 0.229) (**Figure 1**). For women, whites had the worst OS among all racial groups (P < 0.0001); for men, there was no statistically significant difference in survival between different races (figures not shown).

### Survival Analysis by Stage at Diagnosis and Histologic Subtype

**Figure 2:**
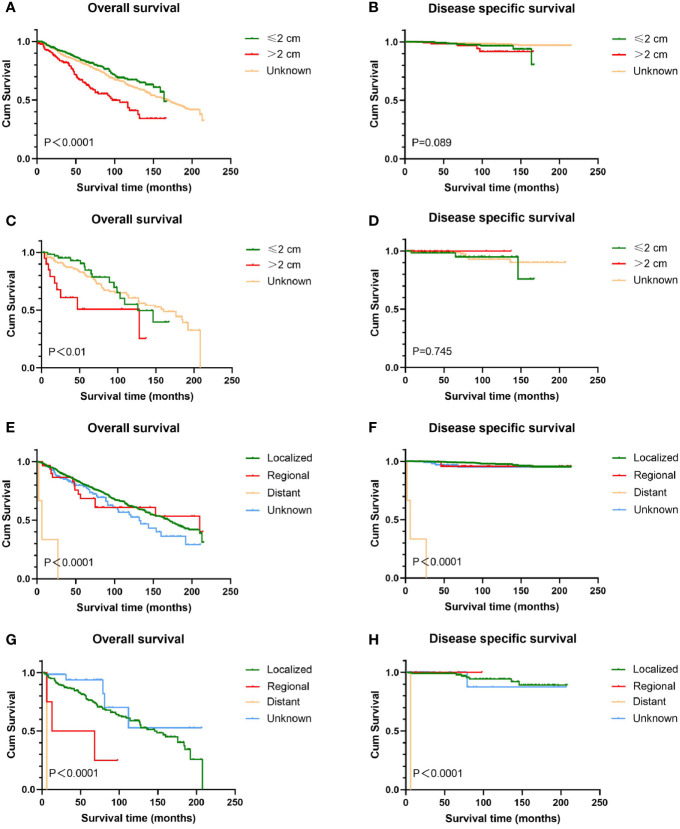
Kaplan-Meier estimates of OS and DSS by tumor size and stage. Patients with tumors >2 cm had worse OS than those with tumors ≤2 cm **(A, C)**; tumor size has no prognostic value for DSS in patients with genital BCC **(B, D)**. Patients with distant disease experienced the lowest survival time **(E–H)**. Male with localized disease had relatively high OS than those with regional disease **(G)**, while there was no significant difference in DSS **(H)**. Female with localized and regional disease had similar prognosis **(E, F)**. OS, overall survival; DSS, disease-specific survival; BCC, basal cell carcinoma.

In this study, 1,252 cases with known SEER stage were available, and only five patients were distant disease, accounting for 0.4%. All patients with distant disease died of genital BCC and experienced the lowest survival time (average 8.2 months). For men, patients diagnosed with localized disease experienced relatively high OS (mean 131.6 months) than patients with regional disease (mean 46.3 months). No significant difference in both OS and DSS was found among women with localized and regional disease (**Figure 2**). Regarding histologic subtype, there were no significant survival differences (figures not shown).

### Survival Analysis by Tumor Size and Anatomic Site

**Figure 3:**
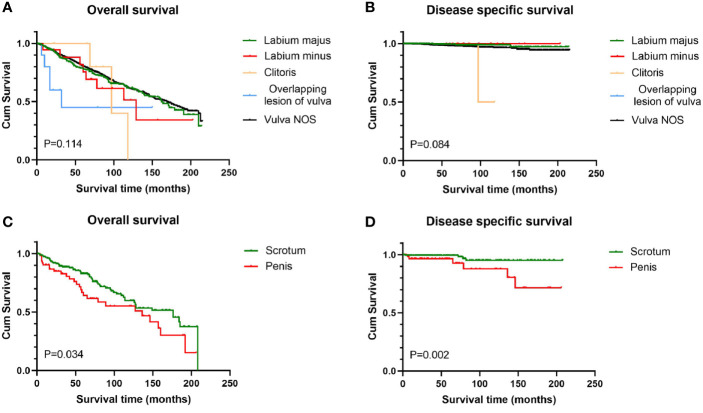
Survival analysis of genital BCC by subsite. Scrotum BCC had statistically better OS and DSS than penis BCC **(C, D)**; for female genital BCC, anatomic site had little prognostic value **(A, B)**. OS, overall survival; DSS, disease-specific survival; BCC, basal cell carcinoma.

Survival analysis from Kaplan-Meier curves revealed that patients with tumors larger than 2 cm had worse OS than those with tumors smaller than 2 cm, regardless of gender, while there was no statistical difference in DSS between the two groups (**Figure 2**). In terms of anatomic site, BCC located on the scrotum had statistically better survival rates than that on the penis, no matter OS or DSS (P < 0.05 for both endpoints). Contrary to male genital BCC, anatomic site had no prognostic value in female survival (**Figure 3**).

### Multivariate Analysis on OS

After accounting for other factors including race, primary site, tumor stage, and tumor size, multivariate analysis revealed that age at diagnosis was a statistically significant predictor of OS for both women and men. Increasing age had a significant effect on death, with HRs ranging from 3.25 to 16.58 for men, and 2.38 to 25.9 for women. For women, tumor size more than 2 cm (hazard ratio [HR] 1.53 [95% CI 1.11–2.10]) and SEER stage at “distant” (HR 10.24 [95% CI 3.22–32.59]) were associated with worse OS; race “others” and “unknown” were associated with better OS (HR 0.40 and 0.12, respectively) (**Table 3**). For man, anatomic subsite and stage were the determinants of survival. Scrotum BCCs had a better prognosis than BCCs located on penis, and the risk of overall death for penis BCCs was almost 1.8 times (HR1.76 [95% CI 1.06–2.92]) as high as that for scrotum BCCs. Compared to patients with localized disease, patients with regional disease (HR 3.77 [95% CI 1.12–12.71]) and distant disease experienced significantly higher overall mortality (HR 62.04 [95% CI 11.1–346.9]) (**Table 4**). Histologic subtype was not a significant variable for both women and men.

**Table 3:** Multivariate analyses of overall survival for female genital basal cell carcinoma.

Characteristics	HR	95% CI	P-value
Age			
≤60	Reference		
61–70	2.38	1.30–4.34	0.005
71–80	7.08	4.20–11.93	0.000
>80	25.9	15.47–43.38	0.000
Race			
White	Reference		
Black	0.69	0.31–1.56	0.377
Others	0.40	0.24–0.70	0.001
Unknown	0.12	0.02–0.87	0.036
Tumor size(cm)			
≤2	Reference		
>2	1.53	1.11–2.10	0.009
Unknown	1.27	1.00–1.62	0.055
Primary site			
Labium majus	Reference		
Labium minus	1.66	0.80–3.47	0.176
Clitoris	1.11	0.35–3.50	0.865
Overlapping lesion of vulva	1.88	0.76–4.65	0.171
Vulva NOS	0.88	0.71–1.11	0.282
SEER Historic Stage A			0.112
Localized	Reference		
Regional	1.12	0.64–1.97	0.685
Distant	10.24	3.22–32.59	0.000
Unknown	1.27	0.94–1.71	0.117

**Table 4:** Multivariate analyses of overall survival for male genital basal cell carcinoma.

Characteristics	HR	95% CI	P-value
Age			
≤60	Reference		
61–70	3.25	1.15–9.18	0.026
71–80	5.51	2.03–14.94	0.001
>80	16.58	6.24–44.04	0.000
Race			
White	Reference		
Black	0.38	0.05–2.90	0.351
Others	0.75	0.18–3.17	0.697
Unknown	0.40	0.05–2.90	0.362
Tumor size(cm)			
≤2	Reference		
>2	1.38	0.57–3.34	0.478
Unknown	0.70	0.39–1.26	0.230
Primary site			
Scrotum	Reference		
Penis	1.76	1.06–2.92	0.029
SEER Historic Stage A			
Localized	Reference		
Regional	3.77	1.12–12.71	0.032
Distant	62.04	11.10–346.88	0.000
Unknown	0.64	0.26–1.63	0.353

## Discussion

UV-light exposure is the major risk factor in the onset of BCC on sun-exposed areas, and BCC mainly affects Caucasians due to their skin’s weak ultraviolet resistance (5). There was almost no record of nude sunbathing in patients with genital BCC in previous retrospective studies or case reports, the relationship of UV with genital BCC is not yet clear.

It is interesting to note that in this study, a majority of patients diagnosed with genital BCC in the US were Caucasians. Of course, it is necessary to further calculate the relative incidence of each race. We assumed that genetic conditions may play a role in the pathogenesis of genital BCC (6). Other risk factors include depressed immune surveillance caused by UV radiation at distant sites or advanced age (7, 8), local trauma or burn (9, 10), ionizing radiation (11), chronic skin irritation (12), human papillomavirus (HPV) infection (13), and exposure to carcinogens, especially arsenic (14, 15). However, only a small percentage of patients in previous case studies were found to have relevant predisposing factors, and HPV was not detected in most BCC specimens (12). The etiology of genital BCC remains unknown.

Genital BCCs tend to develop in an older age group, with similar mean age at presentation for women (mean 71 years) and men (mean 69.9 years) in this cohort. Male sex is one risk factor for BCC outside the genitalia, while genital BCCs are predominantly reported in females in previous literature (16, 17). Similarly, in this study, the majority of patients with genital BCC were females, and the female to male ratio was 5.3. The reason for this female predominance remains to be clarified. One possible explanation is the presence of a chronic skin irritation such as chronic vulvovaginitis, since most patients with vulvar BCC tend to be postmenopausal with drastically reduced hormone levels (16). Despite the higher incidence, females had better DSS than males, and the cause remains to be elucidated.

Most BCCs are indolent, only infiltrate locally, and rarely metastasize (18). As a result of delayed visits and diagnosis, the tumor size of genital BCCs tends to be large. Patients may hesitate to seek treatment because of the privacy of genital area. In addition, the presentation of vulvar BCC is variable and nonspecific, such as itching, a lump, bleeding, and pain (19, 20). Physicians may initially mistake lesions for inflammatory or infectious dermatoses (21). In this study, there were 718 cases with known size (mean 1.77 cm), and tumors greater than 2 cm accounted for 27.2%. For women, tumors over 2 cm conferred worse prognoses (HR 1.53 [95% CI 1.11–2.10]) than those less than 2 cm on multivariate analysis. Only four cases in this cohort had distant metastasis; all of them died of disease-specific death with survival ranging from 0 to 27 months (average 9.8 months). Thus, although rare, genital BCC still has the risk of distant metastasis, and the prognosis is very poor once it occurs. Additionally, the skin lesions on the genital area should be differentiated from malignancy such as squamous cell carcinoma, malignant melanoma, Bowen’s disease, and extramammary Paget (22). It is recommended that all suspect lesions on the genital skin be biopsied as soon as possible to confirm the diagnosis and improve the prognosis.

The major histologic presentation of genital BCCs in both men and women was nodular subtype, which was similar to non-genital BCC (23). In this study, there was no statistical difference in survival between histologic subtypes by Kaplan-Meier survival analysis and multivariate analysis. According to the World Health Organization’s coding and classification of BCC, different histologic subtypes share the same code (such as 8097/3 for micronodular and nodular subtype), which may affect the results of survival analysis to some extent. Additionally, we did not include basal cell adenocarcinoma in our study due to its low incidence in the genital area and the small number of cases in the SEER database. Mitchell has conducted a population-based survival analysis on basal cell adenocarcinoma, while patients with the involvement of penis, scrotum, and vulva accounted for only 0.4, 0.8, and 10.1%, respectively. Therefore, it is difficult to obtain survival data of genital BCC from this research.

Consistent with previous reports (24), in this cohort, most vulvar BCCs occurred on the labia majus, accounting for 89.6% of all vulvar BCC cases with known explicit location. BCCs of the penis were less frequently reported than BCCs of the scrotum (25), and in this cohort, the total number of penis BCCs accounted for only one-third of the scrotum BCCs. We found that penis BCCs showed poorer prognosis than scrotum BCC, with about 1.8 times higher risk of death. In addition, only two male patients with distant disease were identified, and all occurred on the penis. To our knowledge, this is the first comparison conducted on genital BCCs between penis and scrotum, and further research is needed to verify this conclusion.

Our study does have some limitations. Several known factors such as margin of resection, surgical modalities, depth of invasion, genetic factors, comorbid conditions, systemic treatment, and the time interval from onset of symptoms to diagnosis are not available in the SEER database. These variables are generally considered predictors of prognosis. BCC is an indolent skin malignancy with high recurrence but few metastases. Given that the SEER database does not contain information regarding disease recurrence, we cannot determine recurrence rates and recurrence-free survival. In addition, we were unable to calculate the metastasis rates and identify metastasis-related risk factors due to the missing and obscure information on metastasis. Therefore, it is difficult to conduct in-depth research to determine whether the biological behavior of genital BCCs is more aggressive than BCCs located on extragenital skin.

As mentioned previously, different histologic subtypes share the same code, causing misclassification in this study. Furthermore, the primary site coding was ambiguous (i.e., “Overlapping lesion of vulva” and “Vulva NOS”), and data on tumor size and stage are largely missing, so these patients could not be included in specific analysis by site, size, and stage, respectively. In addition, the SEER database does not specify the age of patients over 85 years, and we replaced it with 85 in calculation, which may lead to an underestimation of the average age. It is important to emphasize that our research was based on US population data, and our results may not be universally applicable to the world. Finally, these data are retrospective and prospective research is still necessary to further verify the results of this study.

Despite the above limitations, this population-based study represents the first large-scale analysis of genital BCC, providing more insight and comprehensive understanding into this rare skin cancer, and encouraging follow-up research.

## Conclusion

This study offers a comprehensive analysis on genital BCC from a well-defined population. In general, genital BCCs have great prognosis, but the survival becomes very poor once distant metastasis occurs. Despite similar clinicopathologic features and OS outcomes, men and women should be treated as two different entities when making survival predictions.

## Data Availability Statement

The datasets presented in this study can be found in online repositories. The names of the repository/repositories and accession number(s) can be found below: (http://www.seer.cancer.gov).

## Ethics Statement

Ethical review and approval was not required for the study on human participants in accordance with the local legislation and institutional requirements. Written informed consent for participation was not required for this study in accordance with the national legislation and the institutional requirements.

## Author Contributions

XC drafted the manuscript. YH participated in the design of the manuscript and made relevant statistical analysis. CC collected the related literature. All authors contributed to the article and approved the submitted version.

## Conflict of Interest

The authors declare that the research was conducted in the absence of any commercial or financial relationships that could be construed as a potential conflict of interest.
